# Esthetic Rehabilitation of Anterior Teeth with Laminates Composite Veneers

**DOI:** 10.1155/2014/849273

**Published:** 2014-06-11

**Authors:** Dino Re, Gabriele Augusti, Massimo Amato, Giancarlo Riva, Davide Augusti

**Affiliations:** ^1^Department of Oral Rehabilitation, Istituto Stomatologico Italiano, University of Milan, 20122 Milan, Italy; ^2^Department of Medicine and Surgery, University of Salerno, 84084 Salerno, Italy

## Abstract

No- or minimal-preparation veneers associated with enamel preservation offer predictable results in esthetic dentistry; indirect additive anterior composite restorations represent a quick, minimally invasive, inexpensive, and repairable option for a smile enhancement treatment plan. Current laboratory techniques associated with a strict clinical protocol satisfy patients' restorative and esthetic needs. The case report presented describes minimal invasive treatment of four upper incisors with laminate nanohybrid resin composite veneers. A step-by-step protocol is proposed for diagnostic evaluation, mock-up fabrication and trial, teeth preparation and impression, and adhesive cementation. The resolution of initial esthetic issues, patient satisfaction, and nice integration of indirect restorations confirmed the success of this anterior dentition rehabilitation.

## 1. Introduction

New ceramic and composite materials have increased conservative treatments of compromised anterior teeth [1, 2]. Indirect additive veneering was introduced in the 1980s as an alternative to full-coverage crowns. The concept of no-preparation or minimal-preparation [3] has followed the development of appropriate enamel bonding procedures. The color and integrity of dental tissue substrates to which veneers will be bonded are important for clinical success [4]; using additional veneers with a thickness between 0.3 mm and 0.5 mm, 95% to 100% of enamel volume remains after preparation and no dentin is exposed [5]. A number of clinical studies have concluded that bonded laminate veneer restorations delivered good results over a period of 10 years and more [6–8]. The majority of the failures were observed in the form of fracture or marginal defects of the restoration [9]. Pure adhesive failures are rarely seen when enamel is the substrate with shear bond strength values exceeding the cohesive strength of enamel itself [10, 11]. Some indications for no-preparation veneering include erosion, incisal edge microfractures, corrections for short and small crowns (particularly in patients with larger lips), and alterations in the superficial enamel texture. Restoration of missing dental tissue with resin composites is quick, minimally invasive, and inexpensive and the resulting restorations are easy to repair, if necessary [12]. The present case report describes the treatment of wear in the anterior dentition with thin composite laminate veneers, to restore esthetics and function.

## 2. Diagnosis and Treatment Planning

**Figure 1:**
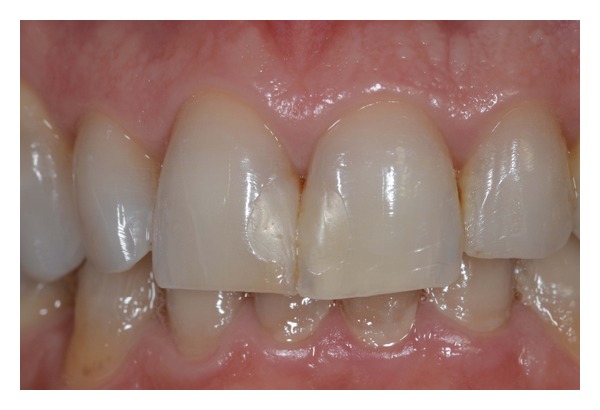
Intraoral anterior view of teeth before treatment.

A 41-year-old male patient was concerned about his smile; clinical examination revealed unsatisfactory composite restorations of upper central incisors, a discrepancy in incisal marginal levels, and an asymmetry between lateral incisors. Previous orthodontic treatment did not completely solve the right lateral incisor rotation (Figure 1).

**Figure 2:**
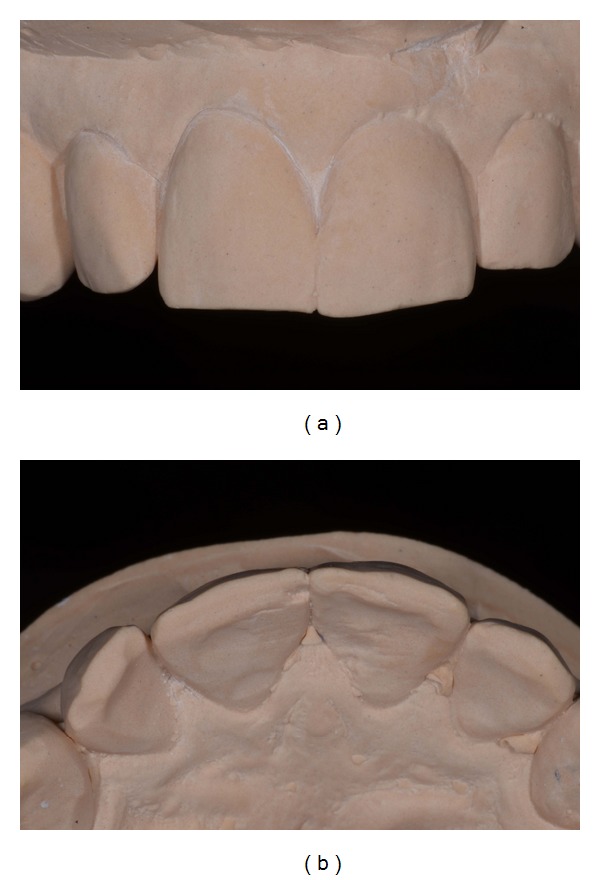
(a) Preoperative cast. (b) The same cast with horizontally sectioned silicon index of wax-up.

During the first appointment a complete radiological and photographic documentation was collected; an alginate impression was taken for the preliminary wax-up and mock-up procedures. Impressions were poured in type IV dental stone; the models were mounted on a semiadjustable articulator (Figures 2(a) and 2(b)).

**Figure 3:**
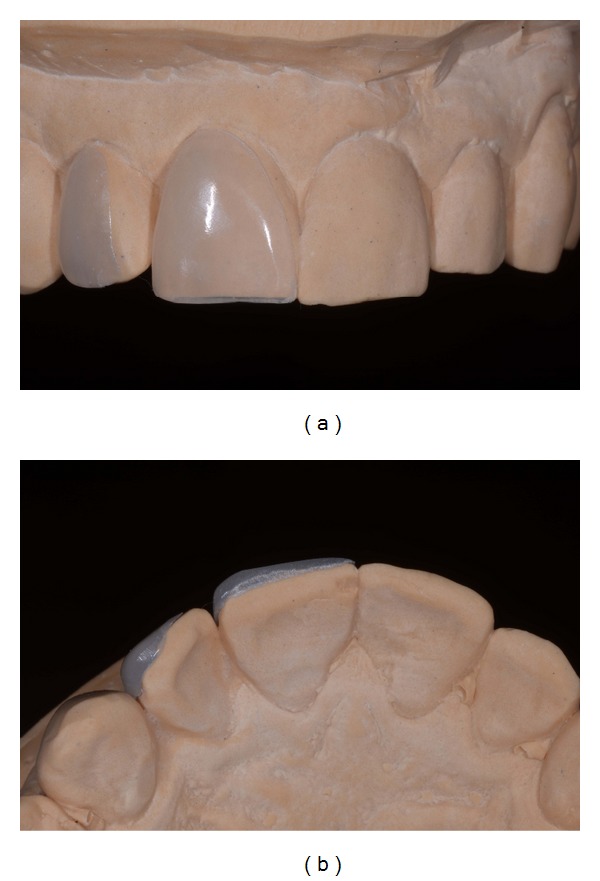
(a) Frontal view of the mock-up made in PMMA resin of teeth 11-12. (b) Occlusal view of the same mock-up.

The preliminary mock-up of teeth 12 and 11 was performed by the technician using bisacrylic resin (Protemp 4, 3M ESPE) to simulate the final result, determining an ideal width and length teeth ratio (Figures 3(a) and 3(b)).

**Figure 4:**
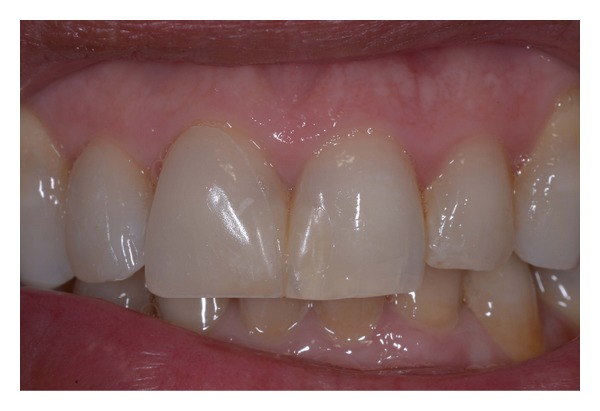
Intraoral try-in of the mock-up and provisional cementation.

During a second appointment the preliminary resin mock-up was tried, checked, and provisionally luted (Figure 4); any alteration desired by the patient and the clinician was analyzed, discussed, and adjusted. Static and dynamic dentofacial aspects were evaluated, considering lip line and maxillary teeth exposure. The final treatment plan was approved by the patient after form and function were confirmed as well.

**Figure 5:**
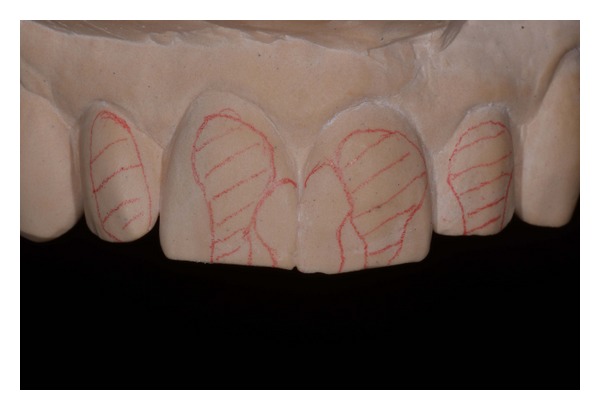
Minimal tooth preparation areas marked on the preoperative cast.

After all information was obtained by the mock-up, the technician checked the space available for the veneers and the path of insertion and highlighted on the dental stone model the areas requiring clinical preparation (Figure 5). A silicon index was also provided for the clinician in order to evaluate the veneers space requirements.

## 3. Dental Preparation

**Figure 6:**
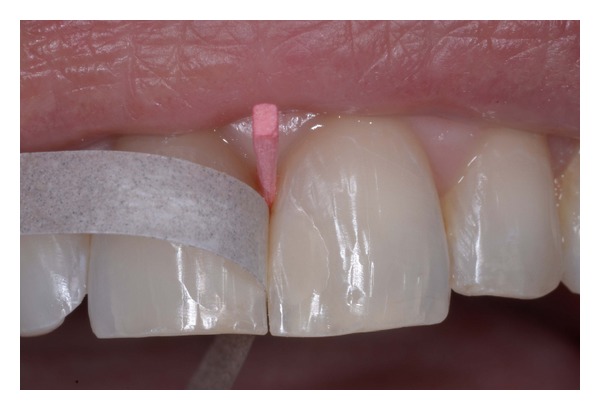
Interproximal preparation using finishing strip.

The least invasive preparation with maximal preservation of enamel was performed. In order to facilitate the impression and cementation protocols, interproximal space between central incisors was at first widened using coarse/medium finishing strips (each of them has two different abrasive grades) (Sof-Lex, 3M ESPE); subsequent surface refinement was performed by fine/superfine strips of the same system (Figure 6). Medium grit (100 *μ*m) and fine grain (30 *μ*m) tapered diamond burs (number 868.314.016 and number 8868.314.016, Kit 4388, Komet Dental, Milan, Italy) were used to reduce the labial surfaces; clearance for the composite restoration was checked with the silicon guide.

**Figure 7:**
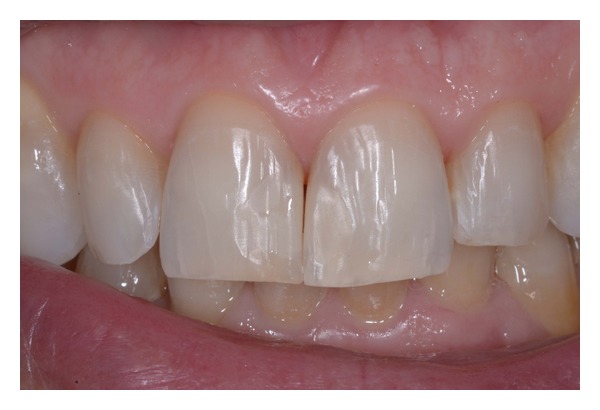
Refined and polished surfaces before final impression.

All the teeth surfaces and past composite restorations were finished using stone burs (based on a micrograined aluminum oxide grit; Dura-white stones, Shofu Dental GmbH, Ratingen, Germany) and polishing disks (Sof-Lex Extra Thin (XT) disks, number 2382SF and number 2382F, 3M ESPE) to promote maximum impression material adaptation and adhesive cementation (Figure 7).

## 4. Chairside Impression

**Figure 8:**
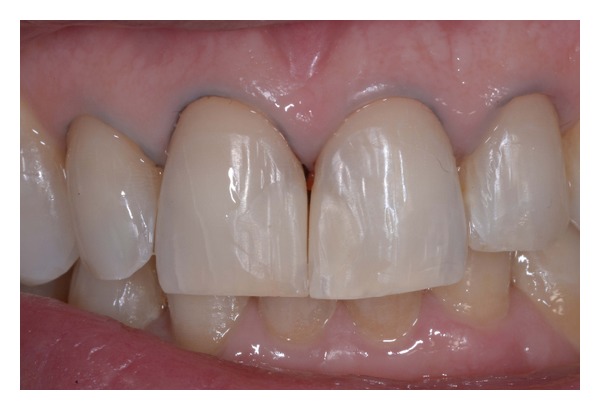
Ultrapak retraction cord in situ.

Following the preparations, a small diameter retraction cord was placed in the bottom of the sulcus to obtain an adequate gingival displacement (number 00 Ultrapak, Ultradent Inc.). The cord was left in the sulcus during the entire impression procedure; this technique limits the flow of crevicular fluid and provides correct moisture control (Figure 8).

**Figure 9:**
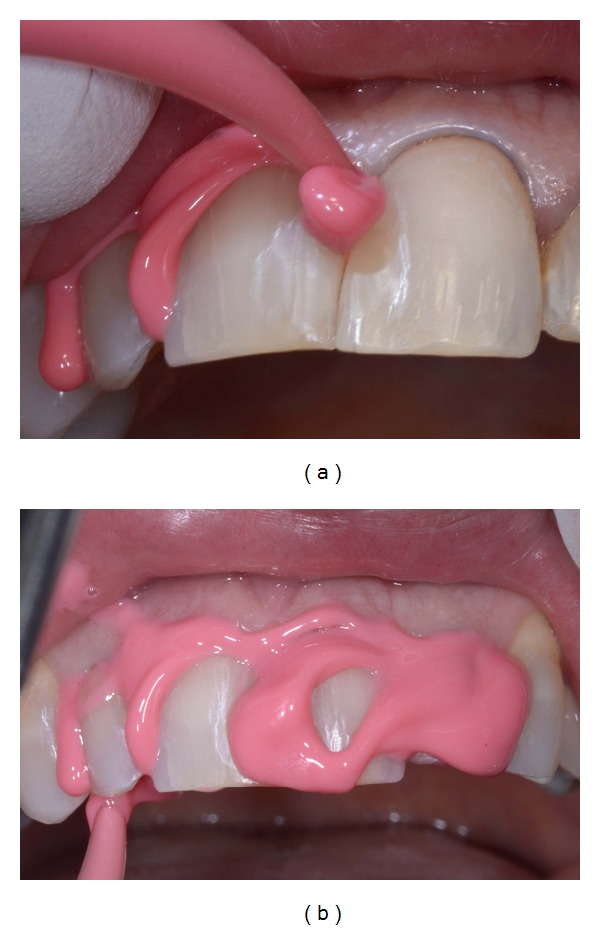
(a)-(b) Impregum Duosoft light body material injection.

**Figure 10:**
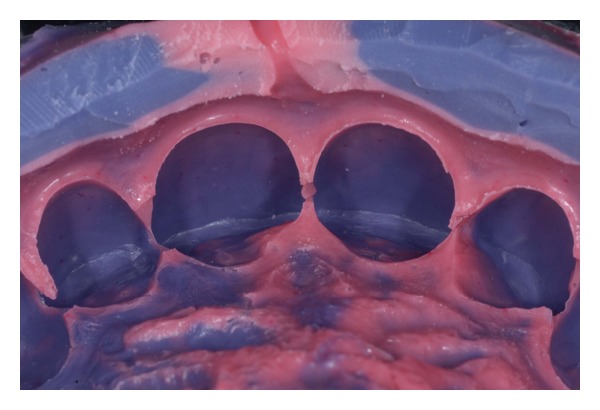
Detail of the final elastomeric impression.

A high quality polyether precision material was used (Impregum Penta Duosoft, 3M ESPE) to take a one-step, double mix final impression; a light body (Duosoft L, 3M ESPE) was applied at the gingival margin and gently blown over the preparations (Figures 9(a) and 9(b)). A full metallic tray was loaded with the heavy body impression material (Duosoft H, 3M ESPE), inserted in the oral cavity, and allowed to set according to the manufacturer's instructions and then removed (Figure 10).

## 5. Laboratory Fabrication of Veneers

**Figure 11:**
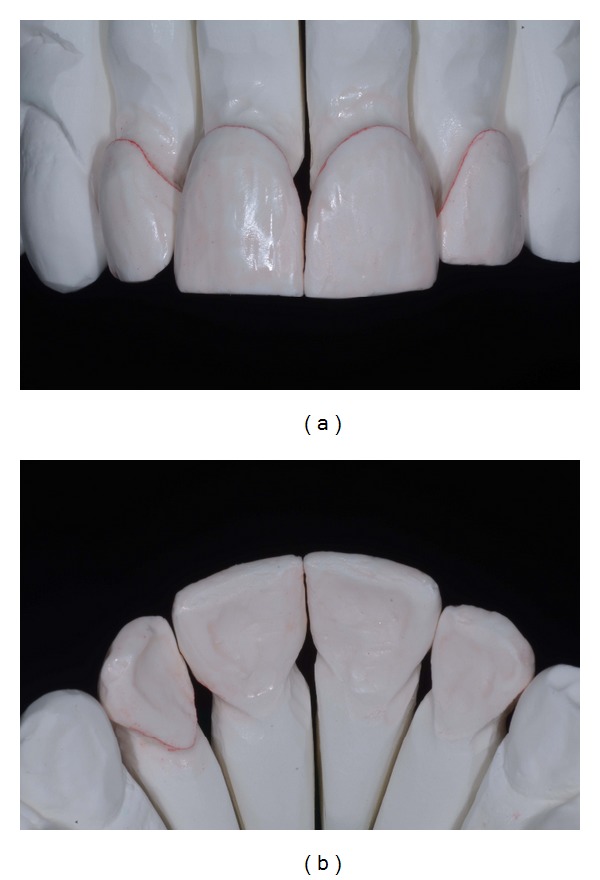
(a)-(b) Buccal and palatal view of the upper ditched master cast.

**Figure 12:**
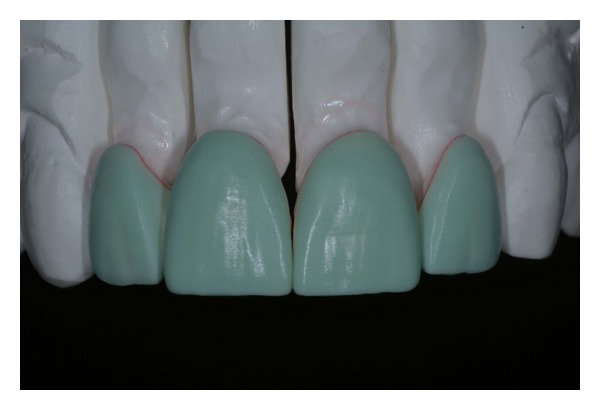
Wax-up for definitive restorations.

**Figure 13:**
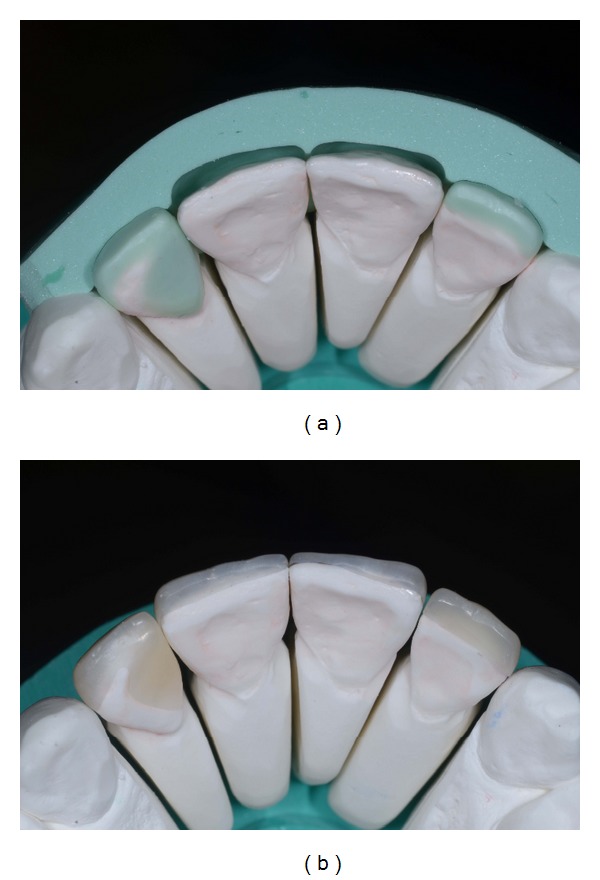
(a) Wax-up silicon index. (b) Microhybrid resin composite veneers fabrication.

**Figure 14:**
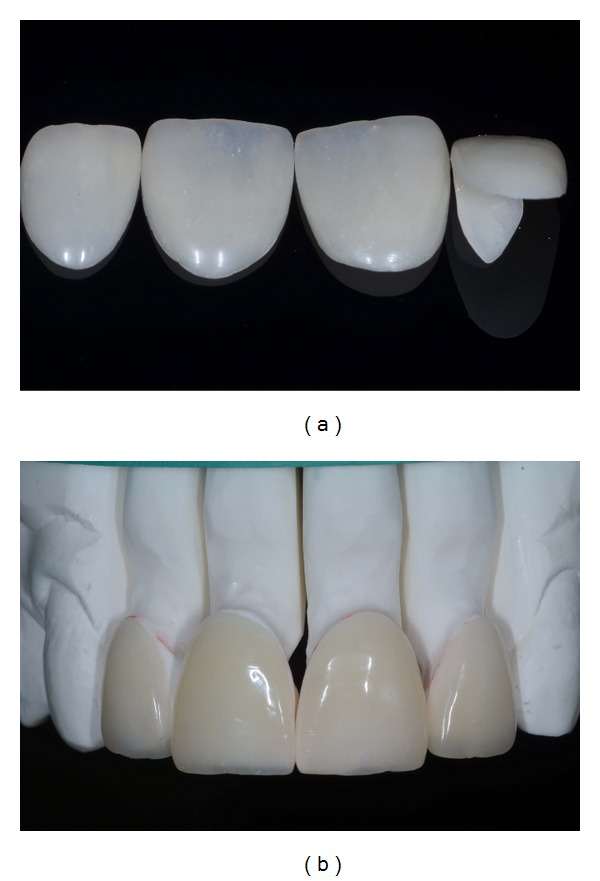
(a)-(b) Finished and polished restorations.

A working model of the upper arch was obtained by pouring the definitive elastomeric impression (Figures 11(a) and 11(b)). The dental technician prepared a wax-up on the working cast, reproducing all diagnostic information previously approved by the patient (to accomplish this step, the clinician recorded and sent to the laboratory an alginate transfer impression of the mock-up in situ) (Figure 12). A microhybrid with nanoparticles resin material (Miris 2, Coltène Whaledent) was positioned at the inner surface of a silicon index and composite restorations of the four upper incisors were baked on the working model (Figures 13(a) and 13(b)). While for teeth 11, 21, and 22 vestibular and incisal areas were covered by the veneers, for the upper right lateral (tooth 12) the mesiopalatal surface was also involved. No dye spacer was used on dental cast so as to achieve optimal adaptation of the restoration with minimal thickness of resin composite cement. Microtextures detailing was accomplished with finishing diamond burs. Composite restorations were finally refined: the adjustment of rough contours and polishing with stones (Dura-green stones, Shofu Dental GmbH, Ratingen, Germany) and disks (Sof-Lex, 3M ESPE) allowed the fabrication of life-like veneers (Figure 14(a)). Definitive restorations were seated on the working model and sent to the clinician (Figure 14(b)).

## 6. Luting

**Figure 15:**
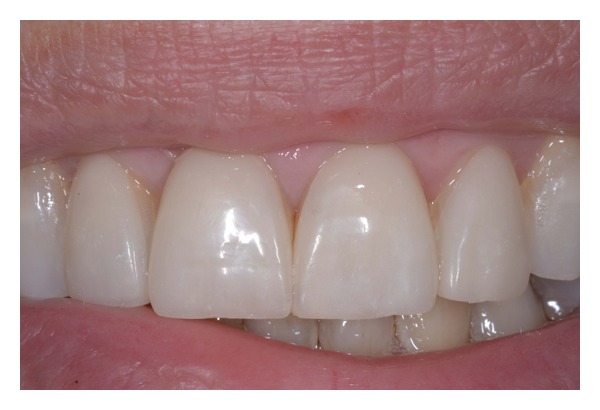
Evaluation of the shade and fit using try-in paste.

**Figure 16:**
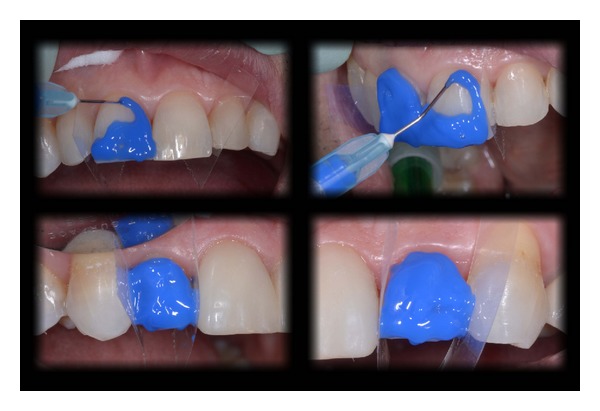
Phosphoric acid (38%) etching of enamel (30 s).

After one week the patient returned for placement of the final veneers and a try-in was carried out. The teeth were cleaned with pumice and dried and a transparent try-in paste was applied on the intaglio surface (Variolink try-in paste, Ivoclar) (Figure 15). The marginal adaptation was checked with a probe using dental loupes (Orascoptic HiRes 3.3x magnification). An adhesive cementation was performed: the dental enamel surface and the inner veneer surfaces were treated before luting. The first one was etched with 38% phosphoric acid for 30 seconds, washed for 60 seconds, and gently dried (Figure 16); then a universal dental adhesive (Scotchbond Universal, 3M ESPE) was applied using a microbrush.

The inner surface of the sectional veneers was sandblasted with 50 micron Al_2_O_3_ for 10 seconds at 2.8 bar pressure; the indirect restorations were ultrasonically cleaned to remove any remnants of alumina particles. A silane coupling agent (ESPE-Sil, 3M ESPE) was used to facilitate the creation of high bond strength to the cement. A coat of adhesive was applied to the inner surface of the restorations and left uncured. All surface treatments illustrated are reported in Table 1.

A thin layer of preheated resin composite material was used as the luting agent (Miris 2, Coltène Whaledent) and directly applied to the inner surface of veneers. Restorations were slowly seated on their respective teeth preparations; pressure was applied in order to facilitate adaptation and flow of the luting agent. While handling the veneers in place, excess resin cement was carefully removed using a sickle-shaped scaler (Novatech cement remover, Hu-Friedy Co., Chicago, USA). Glycerine gel was applied at the margins to prevent an oxygen inhibition layer at the interface; subsequently a prolonged light curing was performed at facial, incisal, and palatal sides for 90 seconds each (Bluephase LED curing light, Ivoclar). The entire cementation procedure was performed in two steps: first on central incisors and then repeated on laterals. Following photopolymerization, residual remnants of cement were removed with the help of a number 12 surgical blade and a dental probe; flossing was performed at the interproximal areas to confirm patency at the contact points. Margins were finished and polished with diamond burs, rubber points, and diamond polishing paste.

## 7. Esthetic Result

**Figure 17:**
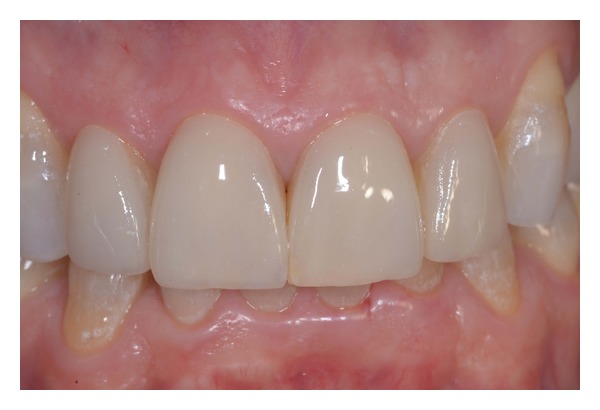
Postoperative intraoral view.

**Figure 18:**
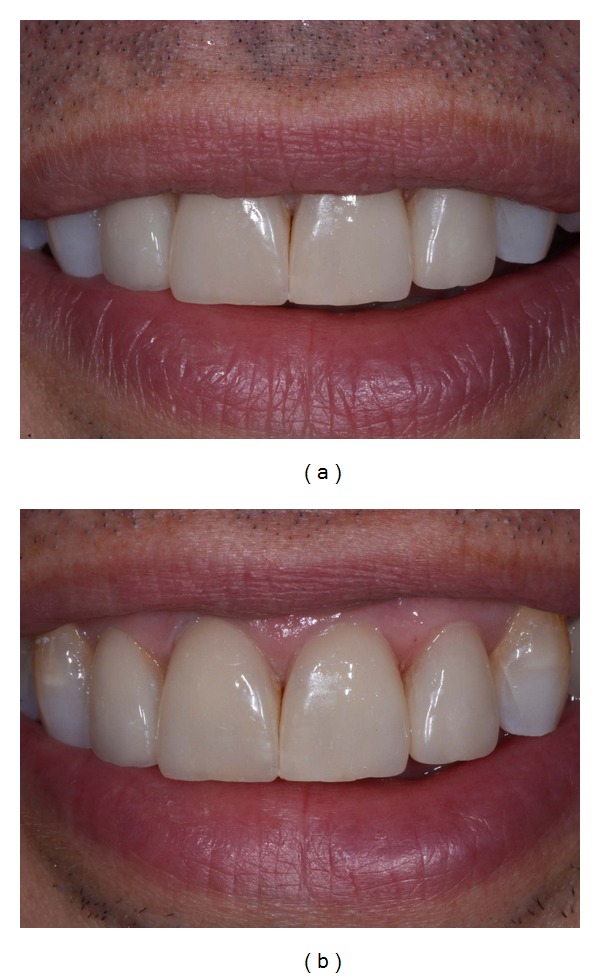
(a)-(b) Postoperative dentolabial views; smile integration of final work.

Intraoral and dentolabial views of the postoperative smile enhancement at the follow-up, one week after the cementation procedure, are shown in the figures (Figures 17, 18(a), and 18(b)); the final result met the patient's expectations. The obtained gingival health status, along with the resolution of initial esthetic issues (in particular, lateral incisor rotation and inadequate composite fillings) and nice integration of indirect restorations, confirmed the success of this anterior dentition rehabilitation.

## 8. Discussion

The cosmetic improvement of the smile is possible with both direct [13, 14] and indirect techniques [12, 15]; the latter procedures might require more than one appointment but are preferred when multiple teeth are involved in the treatment plan and accurate tooth reshaping or color matching is needed [12]. With indirect techniques a previsualization of the final esthetic result is extremely useful both for the clinician and for the patient: in this way, desires and preferences related to the new smile are tested before carrying out irreversible teeth preparations [16, 17]. For these reasons, a diagnostic approach is highly recommended when interventions are focused on the anterior area of the mouth. In this case report, the gingival outline of anterior teeth was considered satisfactory; in other situations previsualization templates are also useful to plan and/or carry out soft tissue recontouring, conditioning, and/or gingivectomy [18]. While in the past full-crowns were indicated in similar clinical scenarios, the improvement in adhesive technologies has made possible a variety of more conservative treatments. Different materials could be used to fabricate additional veneers: feldspathic ceramics [2], hybrid composites [12], or high-density ceramics (alumina, glass-infiltrated zirconia, zirconia) [19]. The last, which is usually CAD/CAM processed, needs more improvements in the anterior area for translucency properties; moreover, an optimal adhesive cementation protocol is not yet available [20, 21]. From a biological point of view, margins of indirect veneers are most frequently placed at the juxta or supragingival area; it has been documented that an extrasulcular location is able to provide adequate soft-tissue health [22].

In this case report, a composite material has been selected due to its quick and inexpensive delivering; a nanohybrid composite with nanoparticles was preferred to porcelain due to the ease of handling in the try-in and luting procedures. Fractures of resin composite materials could also be simply repaired with direct chairside techniques [23]. A preheated light-cure composite has been suggested for cementation and selected in our rehabilitation due to the ultrathin thickness of laminates and for its low polymerization shrinkage and coefficient of thermal expansion compared to currently available resin luting agents [24]. Among the disadvantages or resin materials, some concerns have been raised regarding cytotoxicity [25]; however, this might not represent a problem when no direct contact with living cells (i.e., pulp tissue) exists. While the scientific literature is more extensive for ceramic laminates, a recently published clinical trial (with a 3-years follow-up) has reported no significant difference in the survival rate of composite (87%) and ceramic (100%) veneers; on the other hand, some surface quality changes were more frequently observed for the resin materials (i.e., minor voids and defects and slight staining at the margins) [26]. In addition, the survival rate and clinical performance of composite or ceramic laminate veneers were not significantly influenced when bonded onto intact elements or onto teeth with preexisting composite restorations (when no caries or infiltration is present, of course) [27, 28]. As a final recommendation, like after the delivering of many other types of indirect restorations, the dentist should plan a careful follow-up program and give patients appropriate instructions for maintenance and preservation of the obtained success.

## 9. Conclusions

The delivered treatment with resin composite additional veneers followed the principles of enamel preservation to achieve an esthetic rehabilitation of the four upper incisors; the presented case report was based on an accurate diagnostic process (wax-up and in vivo mock-up) which allowed for minimally invasive, selective reduction of tooth substance.

## Figures and Tables

**Table 1:** Conditioning sequences for tooth and restoration surfaces.

Composite veneers pretreatment	
1	Sandblasting with 50 micron aluminum oxide particles (2.8 bar, 10 s, 1 cm)
2	Ultrasonic bath in ethanol (5 min)
3	Silane coupling agent application and evaporation (1 min)
4	Adhesive application (no photopolymerization)
5	Preheated resin composite application on the inner surface of the veneer

Enamel surface pretreatment	
1	Pumice cleaning of teeth surfaces
2	Rinsing with water (1 min)
3	Application of Mylar strips around teeth to be conditioned
4	Phosphoric acid (38%) etching of enamel (30 s)
5	Rinsing with water (1 min)
6	Adhesive application (no photopolymerization)
